# When You Hear Hoofbeats, Think of Zebras: An Autobiographical Case Report of Superior Mesenteric Artery Syndrome

**DOI:** 10.7759/cureus.22519

**Published:** 2022-02-23

**Authors:** Rachel A Nyenhuis, Colleen Moran-Bano

**Affiliations:** 1 Medicine, University of Central Florida College of Medicine, Orlando, USA; 2 Clinical Sciences, University of Central Florida College of Medicine, Orlando, USA

**Keywords:** gastrointestinal surgery, gi radiology, pediatric gi surgery, pediatric radiology, autobiographical case report, pediatric gastroenterology, adult gastroenterology, nutcracker syndrome, duodenojejunostomy, superior mesenteric artery syndrome

## Abstract

Superior mesenteric artery syndrome (SMAS) is a rare, potentially fatal condition that presents with nonspecific gastrointestinal symptoms. Patients often improve clinically following treatment, but complete symptom resolution is challenging to attain. This case report describes the author’s 10-year experience with SMAS following the onset of symptoms at the age of 16 years, as well as sequential diagnoses related to her course of SMAS. Treatment outcomes in the literature, as well as the author’s experience with diagnosis and treatment, will be discussed.

## Introduction

Superior mesenteric artery syndrome (SMAS) is a rare gastrointestinal condition characterized by compression of the third portion of the duodenum by the superior mesenteric artery. Compression results in partial or complete small bowel obstruction, leading to symptoms including nausea, vomiting, postprandial epigastric pain, and early satiety [1]. SMAS is most often precipitated by rapid weight loss or recent surgery, combined with a body habitus that predisposes individuals to the development of symptoms [1]. Once initiated, the syndrome perpetuates itself as further weight loss worsens compression of the duodenum [2]. It affects both sexes and can occur at any age but tends to occur most frequently in adolescent and young adult women [1]. An ethnic predisposition has not been identified [1]. 

Treatments for SMAS can include brief attempts at conservative treatment using nutritional rehabilitation, or surgical correction via duodenojejunostomy or, more rarely, ligation of the ligament of Treitz or transposition of the superior mesenteric artery (SMA) [1,3,4]. Treatment is often clinically successful, however complete resolution of symptoms remains challenging to achieve [5].

This case report describes the author’s experience from the onset of SMAS through diagnosis and treatment, as well as her challenges with consequent medical diagnoses and ongoing symptoms following treatment. The goal of this report is to increase clinician knowledge of the timely and appropriate treatment of SMAS, and to educate clinicians about the long-term complications and residual symptoms that can occur following the treatment of superior mesenteric artery syndrome.

## Case presentation

My path to the diagnosis of superior mesenteric artery syndrome began with a simple case of viral gastroenteritis. I developed nausea and vomiting for two days, then began tolerating oral intake. Over the course of the viral infection and the following days of decreased food intake, I declined from a body mass index (BMI) of 19.0 to 17.8, from 29th to 12th percentile for age and sex. While the virally induced nausea and vomiting resolved, over the next two months I experienced decreased appetite, early satiety, and mild nausea that deterred me from eating as voraciously as I previously did. With the persistent loss of appetite, I continued to lose weight which, due to the pathophysiology of SMAS, worsened my symptoms of nausea and precipitated the onset of post-prandial epigastric pain. After three months, I presented to my family physician for a workup.

Multiple diagnostic studies were performed, including gastric emptying scintigraphy and tissue transglutaminase to rule out gastroparesis and celiac disease, respectively. I was placed on a trial of esomeprazole for suspected acid reflux disease with no improvement. Six months after my symptoms began at the age of 16, I was referred to a Pediatric Gastroenterologist for a more in-depth workup. Gallbladder ultrasound, hepatobiliary iminodiacetic acid (HIDA) scan, stool guaiac and ova and parasite tests, complete blood count (CBC) and C-reactive protein (CRP) tests, and upper esophagogastroduodenoscopy (EGD) were performed, all of which were normal. The discussion began that I may be falsifying my symptoms, with one provider suggesting my diagnosis was anorexia nervosa. Another consulting physician believed my symptoms were due to constipation, a symptom I did not present with. These interactions were particularly damaging, as they weakened the trust between me and my physicians and cast doubt on the legitimacy of my experience.

**Figure 1:**
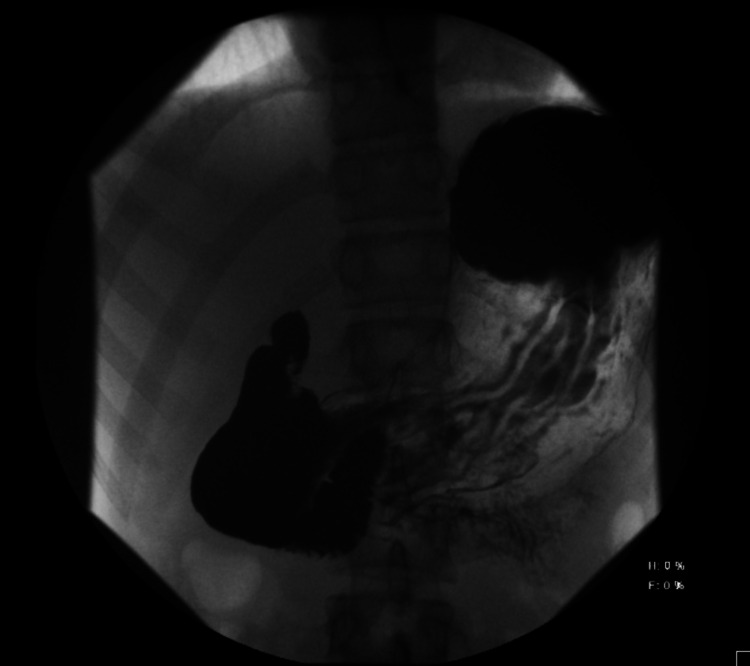
Barium swallow with small bowel follow-through The Barium swallow image is demonstrating filling of the stomach and proximal duodenum without filling of the distal duodenum secondary to obstruction.

**Figure 2:**
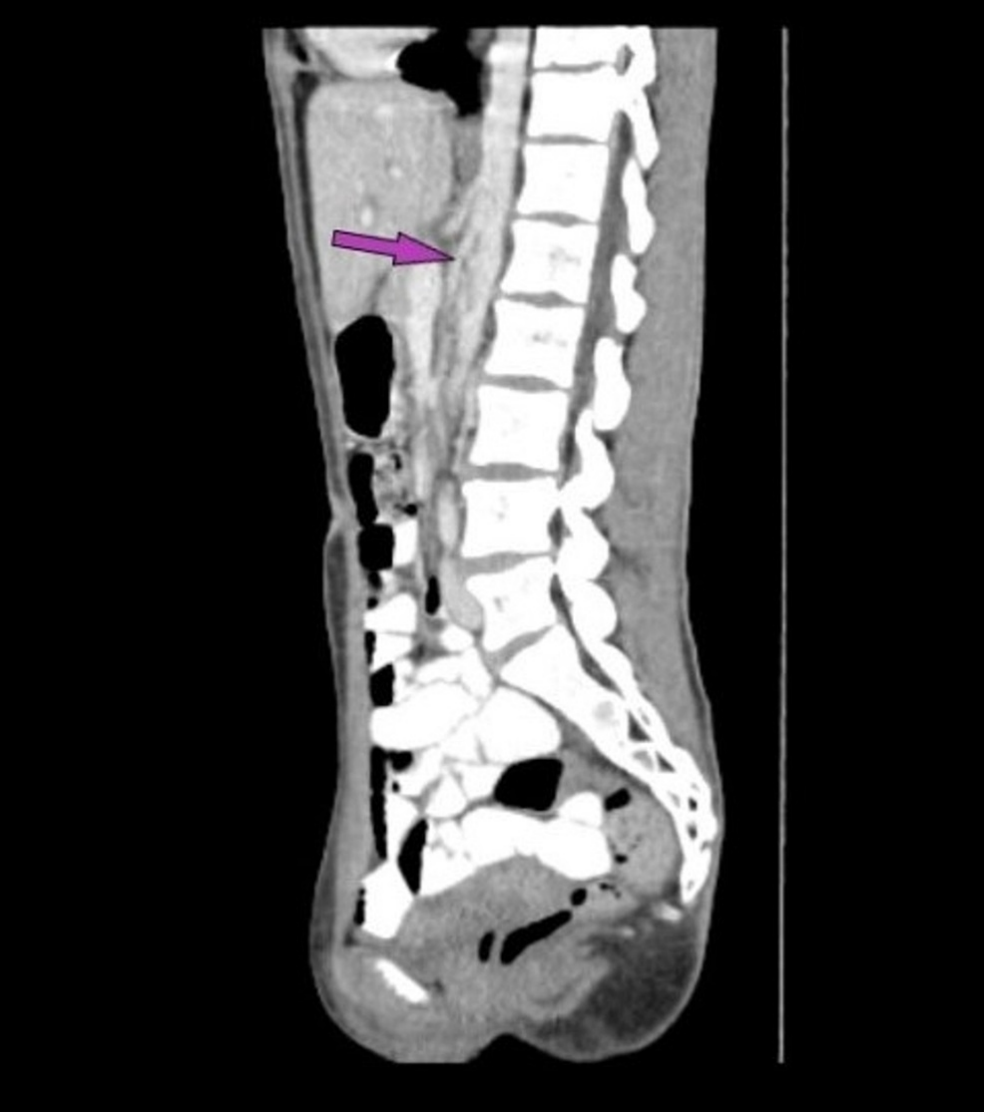
Sagittal contrast-enhanced CT scan demonstrating aortomesenteric angle narrowed to 14 degrees The arrow indicates the superior mesenteric artery.

Fortunately, my gastroenterologist agreed to perform one more diagnostic study to rule out superior mesenteric artery syndrome based on the presence of one distinguishing feature: I felt better when I sat with my knees to my chest, a history finding occasionally noted in SMAS [1]. A barium swallow with small bowel follow-through was performed which demonstrated obstruction of the third portion of the duodenum, consistent with the diagnosis of SMAS (Figure 1). Follow-up contrast-enhanced CT imaging demonstrated an aortomesenteric distance of 5mm and an aortomesenteric angle of 14 degrees (Figure 2). These findings are consistent with previously reported measurements in patients with SMAS, with aortomesenteric distance typically reported between 2-8mm, and aortomesenteric angle reported between 6-22 degrees [6].

Treatment began conservatively, using a high-calorie diet and oral nutritional supplements to reach a healthy BMI, at which point it was believed my symptoms would resolve. This treatment plan failed due to worsening nausea, loss of appetite, postprandial epigastric pain, and right upper quadrant fullness. I also developed severe conditioned food aversions (so-called, “food fear”) due to the close association of eating with the onset of nausea and pain. This significantly affected my ability and willingness to eat sufficiently to gain weight.

Over the next five months, several medications were attempted to address my symptoms, including low-dose amitriptyline for pain, erythromycin to stimulate appetite and intestinal motility, and promethazine and ondansetron for nausea. While helpful, these treatments did not reduce my symptoms adequately to allow sufficient weight gain.

After four months of failed conservative treatment, at the age of 17, I reached a BMI of 14.8 (<1st percentile for age and sex). The decision was made to insert a nasojejunal feeding tube. My weight improved to a BMI of 16.5; however, further weight gain was limited by diarrhea secondary to an intolerable feeding tube rate. The option of surgical treatment was discussed, and with the help of my parents, I made the decision to move forward with an open Roux-en-Y duodenojejunostomy to bypass the compressed portion of the duodenum. I had an uncomplicated recovery and was discharged on postoperative day five.

Following surgical recovery, I had ongoing postprandial epigastric pain and fullness, and nausea, albeit less intense than prior to surgery. These symptoms were initially dismissed by my gastroenterologist. However, after a year without resolution of symptoms and failure to attain a BMI greater than 16.6 (1st percentile for age and sex), further diagnostic studies were performed to identify potential causes of ongoing nausea and abdominal pain. HIDA scan demonstrated a gallbladder ejection fraction of 0%. Laparoscopic cholecystectomy was performed, and pathology results were consistent with chronic cholecystitis. This likely occurred secondary to more than a year of illness and two years of reduced oral food intake. Following cholecystectomy, my nausea greatly improved but did not completely resolve. The surgery had no effect on my epigastric pain.

Shortly after the cholecystectomy, I developed intermittent, intense left-sided pelvic pain, which was not consistent with my previous gastrointestinal symptoms. The pain was worse during menstrual cycles and after moderate to intense physical activity but could occur at any time. A pelvic ultrasound was insignificant, so a trial of oral contraceptives was started for suspected endometriosis. After six months of treatment without symptomatic improvement, I consented to a diagnostic laparoscopy to confirm the diagnosis. Upon laparoscopy, no evidence of endometriosis was found. However, significant congestion of the left ovarian veins was discovered. Considering my previous diagnosis of SMAS, this finding suggested the diagnosis of Nutcracker Syndrome, a condition in which the superior mesenteric artery obstructs the left renal vein leading to renovascular congestion, pelvic and/or flank pain, hematuria, and varicocele in men [7]. Treatment of my pelvic pain using ovarian vein embolization was discussed; however, I declined this intervention, preferring to attempt menstrual cessation via continuous dosing of contraceptives to decrease my pain.

Incidentally, I developed occasional diarrhea and abdominal cramping seven years post-duodenojejunostomy. Diagnostic workup demonstrated lymphocytic colitis. The exact etiology of lymphocytic colitis is unclear; however, it is not believed to be related to any of my previous diagnoses or treatments. These symptoms are well managed with loperamide and lifestyle changes.

At the writing of this case report, I am ten years removed from the onset of SMAS. Clinically, I am much improved. I maintain a BMI of 18.2 (<5th percentile for American adults), near my pre-illness weight. I continue to experience intermittent epigastric pain and right upper quadrant fullness following meals. However, I have learned coping mechanisms over time and can work through the discomfort most days. My nausea has largely resolved, with only a few symptomatic days each month. My left-sided pelvic pain has improved, although I still experience symptoms occasionally. Psychologically, it took years for me to overcome my conditioned food aversions. These improved in part because my nausea has improved. However, there are occasions at which I still feel nervous to eat due to pain; I particularly avoid eating at important events due to the possibility of developing pain.

**Figure 3:**
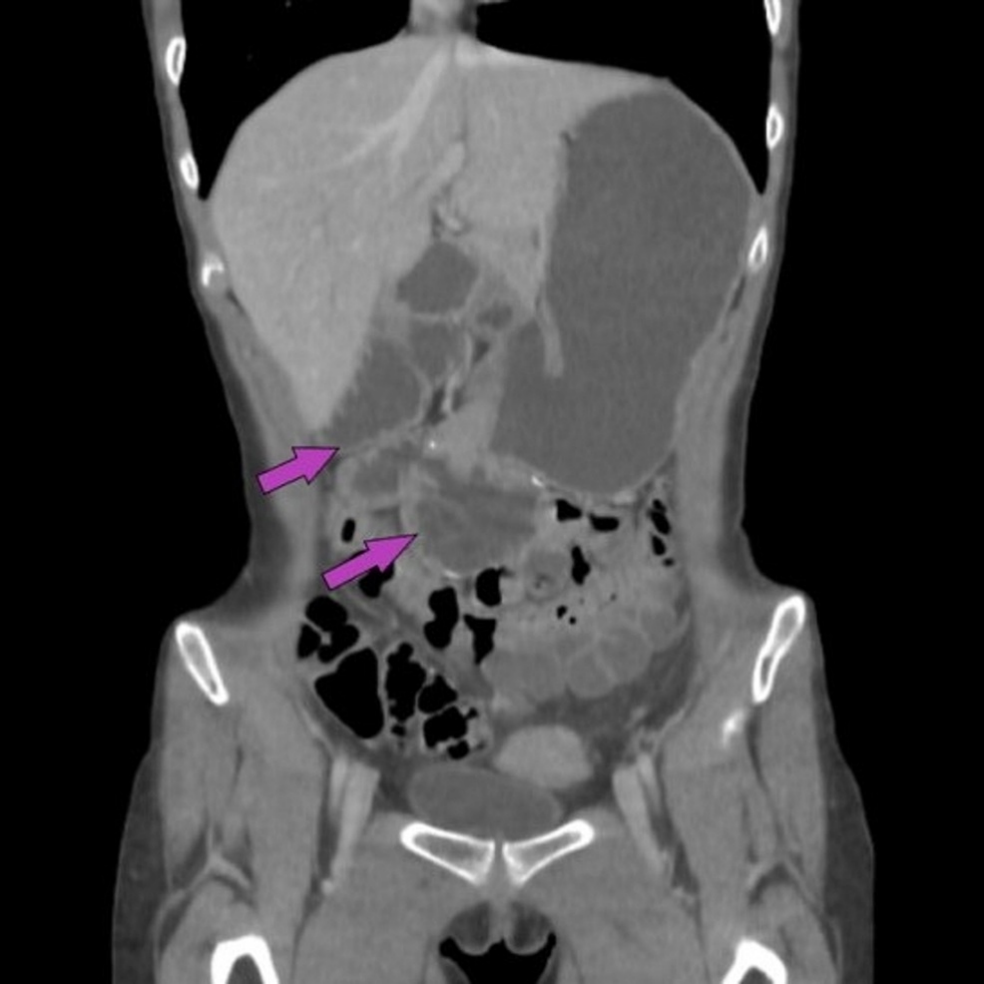
CT enterography demonstrating persistent dilation of the stomach and proximal duodenum six years post-operatively Arrows indicate dilated loops of the proximal duodenum.

My most recent abdominal imaging, performed six years post-operatively, demonstrated ongoing dilation of the stomach and proximal duodenum without evidence of stricture or obstruction (Figure 3). It is unclear why these structures have not returned to a normal volume. It was proposed by my current gastroenterologist, who started treating me in adulthood, that these findings paired with my ongoing symptoms reflect impaired gastrointestinal motility secondary to interruption of the small bowel during the Roux-en-Y procedure. However, I have never undergone small bowel manometry to confirm this theory. I have declined further diagnostic studies at this time due to fatiguing of the testing process and lack of access to a facility with motility-testing capabilities.

My diagnosis with SMAS and the sequential diagnoses fundamentally altered the trajectory of my life. I did not have the pleasure of typical adolescence, as I dealt with the loss of a life-sustaining function, was burdened with daily pain and nausea, and had to learn how to sleep, bathe, and “eat” with a feeding tube. Social events, which often revolve around food, were unenjoyable during this time, as they prompted invasive questions about my condition and heightened feelings of isolation. Prior to my diagnosis, I was active in ballet and lyrical dancing, with the goal of dancing in college. That goal became unattainable as my symptoms precluded the physical strength and endurance necessary to succeed in dance. Despite these challenges and personal losses, it has been a privilege learning to use my experience to help others. As a third-year medical student, I’m poised to treat patients from the perspective of one who has already experienced serious illness. I empathize deeply with the suffering of patients, especially adolescents. Until proven otherwise, I know to take a patient’s word for what they are experiencing, as I understand what it is like to have one’s account doubted. It is a gift to understand the patient perspective early in my training and throughout my career, as it is a skill that cannot be taught except by experience.

## Discussion

Superior mesenteric artery syndrome is usually a diagnosis of exclusion. While some patients experience complete recovery and symptom resolution, it is described in the literature that patients commonly experience ongoing symptoms even after surgical treatment [5]. Unresolved complaints can be related to other comorbidities which confound diagnosis and treatment; however, symptoms have also continued in patients such as myself without prior identifiable comorbid diseases [8,9]. Further research into the ideal diagnostic workup prior to undergoing surgery, and research into the ideal treatment timeline for SMAS, would benefit future patients by informing which treatments will truly benefit each patient. Additionally, more long-term post-operative follow-up studies to identify potential complications which lead to frequent non-improvement following surgery would be beneficial to inform surgical approaches and post-operative treatments. In this author’s opinion, physicians would do well to keep SMAS higher on their list of differential diagnoses in patients presenting with new onset of nausea or epigastric pain, especially in adolescent and young adult patients and those with histories of recent weight loss, illness, or surgery. Early identification of SMAS could prevent the condition from progressing beyond mild to moderate symptoms, allowing more patients to reverse their symptoms via increased oral intake before self-perpetuation of symptoms begins. Once this point is reached, however, evidence supports the use of laparoscopic duodenojejunostomy as an acceptable treatment approach [10,11]. This operation preserves the continuity of the small bowel, benefiting long-term bowel motility, and reduces scarring and pain associated with open approaches. As demonstrated in this report, however, this approach is not a panacea for the treatment of SMAS.

## Conclusions

Superior mesenteric artery syndrome is an uncommon condition that can go unrecognized as symptoms worsen. Prompt diagnosis may prevent the syndrome from progressing to the severity at which it needs surgical intervention. Despite surgical techniques known to improve symptoms, many patients experience ongoing symptoms post-operatively. Further research into the long-term outcomes of patients, as well as the correlation of symptoms with each patient’s treatment timeline and surgical approach may help identify which treatment strategies are most effective and what explanations exist for ongoing epigastric pain and nausea in patients post-operatively.
